# Multicenter comparison of automated procalcitonin immunoassays

**DOI:** 10.1016/j.plabm.2015.07.001

**Published:** 2015-07-17

**Authors:** Mariella Dipalo, Lorena Guido, Gianmatteo Micca, Salvatore Pittalis, Massimo Locatelli, Andrea Motta, Vincenza Bianchi, Tiziana Callegari, Rosalia Aloe, Giorgio Da Rin, Giuseppe Lippi

**Affiliations:** a Laboratory of Clinical Chemistry and Hematology, Academic Hospital of Parma, Parma, Italy; b Clinical Analysis Laboratory, “Cardinal Massaia” General Hospital, Asti, Italy; c Laboratory of Clinical Chemistry and Microbiology, “Maggiore” Hospital, Lodi, Italy; d Laboratory of Clinical Chemistry, “San Raffaele” University Hospital, Milano, Italy; e Laboratory of Clinical Chemistry, “SS Antonio e Biagio e C. Arrigo” Hospital, Alessandria, Italy; f Department of Laboratory Medicine, Hospital of Bassano del Grappa, Bassano del Grappa (VI), Italy

**Keywords:** Procalcitonin, Sepsis, Bacterial infections, Immunoassay, Multicenter study

## Abstract

**Objectives:**

A multicenter study to compare results of BRAHMS Kryptor PCT with those obtained using four BRAHMS-partnered procalcitonin (PCT) automated immunoassays (DiaSorin Liaison, BioMérieux Vidas, Roche Cobas E601 and Siemens Advia Centaur) and the Diazyme immunotubidimetric assay implemented on four clinical chemistry platforms (Abbott Architect c16000, Siemens Advia 2400, Roche Cobas C501 and Beckman Coulter AU5800).

**Design and methods:**

One hundred serum samples from in-patients with PCT values between 0.10 and 58.7 ng/mL were divided into aliquots and tested with the nine different reagents and analyzers. BRAHMS PCT Kryptor results were used as reference.

**Results:**

Compared to BRAHMS PCT Kryptor, significant differences in results were observed on Vidas, Advia Centaur, Architect, Cobas C501 and AU5800. However, the correlation coeffiecients (*r*) with BRAHMS PCT Kryptor were between 0.899 and 0.988. The mean bias was less than ±1.02 ng/mL, except for Vidas (2.70 ng/mL). The agreement at three clinically relevant cut-offs was optimal: between 83–98% at 0.50 ng/mL, 90–97% at 2.0 ng/mL, and 98% at 10 ng/mL. The comparison of Diazyme PCT across the four clinical chemistry analyzers yielded high correlation coefficients (*r* between 0.952 and 0.976), a mean bias less than ±0.9 ng/mL, acceptable agreement at 0.5 ng/mL (>82%), and high concordance at the 2.0 ng/mL (>97%) and 10 ng/mL (>98%) cut-offs.

**Conclusions:**

The methods and applications evaluated in this multicenter study are aligned with BRAHMS PCT Kryptor and can be used for predicting the risk of progression to systemic inflammation in patients with bacterial infections using the conventional PCT diagnostic thresholds.

## 1. Introduction

Procalcitonin (PCT), a 116 amino acid peptide with molecular weight 14.5 kDa, belongs to the calcitonin superfamily of peptides. The protein is encoded by the *CALC-1* (Calcitonin Gene-related Peptide 1) gene on chromosome 11 [1]. The original product of this gene, a 141 amino acid peptide known as pre-PCT, undergoes a proteolytic cleavage of the 25 amino acid signal peptide to produce the PCT molecule, which is then further processed to generate the mature, 32 amino acid-long calcitonin. Under physiological conditions, the transcription of *CALC-1* gene is suppressed in non-neuroendocrine tissues, so that the C cells of the thyroid gland are responsible for the entire synthesis of PCT and calcitonin [1].

In the presence of microbial infections, the synthesis of PCT is dramatically amplified in all parenchymal tissues and differentiated cell types, so that its concentration in blood may increase by several orders of magnitude, up to one thousand-fold [1]. The PCT response to the infection closely mirrors the severity of the inflammatory reaction. More specifically, higher values are associated with severe disease, whereas a declining concentration usually reflects disease resolution [2]. This paradigmatic behavior is now widely used for diagnosis and monitoring of bacterial infections. In particular, PCT values between ≥0.5 and 2.0 ng/mL are suggestive of moderate risk for progression to systemic inflammation, between 2.0 and 10 ng/mL are suggestive for high risk for progression to systemic inflammation, whereas values ≥10 ng/mL are associated with a high probability of developing severe sepsis and septic shock [1]. Recent evidence also suggests that the measurement of PCT may be helpful for the diagnosis of bacterial peritonitis [3], as well as for guiding antibiotic therapy in patients with localized (i.e., community acquired pneumonia) and systemic bacterial infections [4].

Although there is currently no agreed reference technique for PCT measurement, the BRAHMS PCT Kryptor method has been directly developed from the original BRAHMS luminometric immunoassay (LIA), and was the first automated method cleared by the FDA for the diagnosis of severe sepsis and septic shock [5]. Several diagnostic companies such as DiaSorin, BioMérieux, Siemens and Roche have since partnered with BRAHMS for developing automated PCT assays on their proprietary platforms, using different technologies (i.e., immunoluminometric, enzyme-linked immunofluorescent, chemiluminescent and electrochemiluminescent immunoassays). More recently, a latex-enhanced immunotubidimetric assay has also been developed by Diazyme, to be used on a vast array of clinical chemistry analyzers. Nevertheless, due to the lack of a PCT reference material, it remains uncertain whether the current clinical PCT cut-offs would apply to all the available procalcitonin immunoassays, or if monitoring using different methods would be clinically reliable when patients are transferred between healthcare facilities. Therefore, the aim of this multicenter study was to compare results of the BRAHMS Kryptor PCT with those obtained using four BRAHMS-partnered PCT automated immunoassays and the Diazyme immunotubidimetric assay implemented on four different clinical chemistry platforms.

## 2. Materials and methods

### 2.1 Study design

**Fig. 1:**
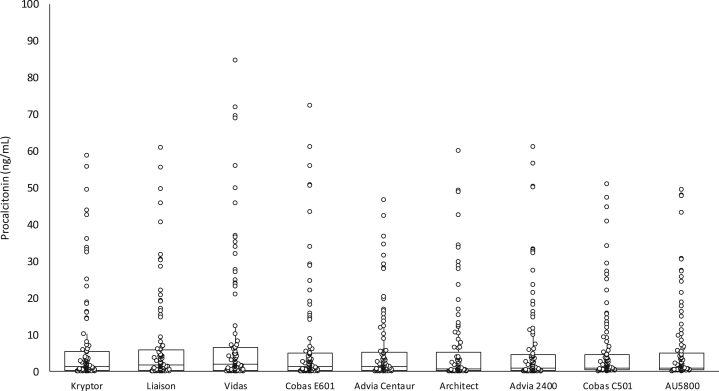
Distribution of procalcitonin values (median and interquartile range) obtained with the different reagents and instruments (see Table 1 for details).

This multicenter study was based on 100 serum samples from inpatients (52 from the intensive care unit, 12 from the emergency department, 15 from the infective diseases unit and 21 from other medical and surgical wards) referred to the laboratory of the University Hospital of Parma for routine PCT measurement during a single working day. The samples were selected to cover the most clinically significant PCT concentrations (0.10–58.7 ng/mL) measured using the local routine immunoassay (BRAHMS PCT Kryptor system, BRAHMS, Hennigsdorf, Germany) (Fig. 1). The blood samples were immediately centrifuged upon arrival in the laboratory, serum was separated and divided into seven aliquots of 0.6 mL each. The first aliquot was used for routine PCT assessment, whereas the remaining six aliquots were frozen at −70 °C. After one week of storage, the aliquots were transported to the participating centers, under controlled conditions of temperature and humidity. Specifically, four reusable dry gel packs containing carbopol gel (2×200 mL and 2×500 mL) were frozen at −70 °C and then placed into certified transport boxes at the sample collection center [6]. The mean time of shipment to the participating centers was 92 min (range: 54–106 min). Upon arrival, the aliquots were stored frozen at −70 °C until all sites had received the shipment. The aliquots were then thawed at room temperature on the same day, and simultaneously analyzed in each center with all reagents and methods, including retesting on BRAHMS PCT Kryptor.

### 2.2 Methods description

The complete description of reagents and instrumentation is shown in Table 1. In brief, the BRAHMS PCT Kryptor system is an immunofluorescent, fully automated and highly sensitive PCT assay. The measurement principle is based on Time-Resolved Amplified Cryptate Emission (TRACE) technology, entailing the measurement of a signal emitted from an immunocomplex with time delay. The functional sensitivity and total imprecision (coefficient of variation, CV) of the assay were previously found to be 0.04 ng/mL and 0.7–8.3%, respectively [7]. Serum procalcitonin was also measured using four BRAHMS-partnered PCT automated immunoassays, the analytical characteristics of which have been previous described elsewhere and are briefly reported in Table 1 [8], [9], [10], [11], [12].

**Table 1:** Description of sites, reagents and instruments used in the multicenter study.

Site	Company	Method	Assay principle	Sample volume (µL)	Measuring range (ng/mL)
Parma, Italy	BRAHMS, Hennigsdorf, Germany	Kryptor BRAHMS PCT sensitive	Time-Resolved Amplified Cryptate Emission (TRACE) immunoassay	50	0.02–1000
Asti, Italy	Diasorin, Saluggia (VC), Italy	Liaison BRAHMS PCT	Two-site immunoluminometric assay	100	0.02–100
Lodi, Italy	bioMérieux, Marcy l’Etoile, France	Vidas BRAHMS PCT	Enzyme-linked fluorescent immunoassay (ELFA)	200	0.05–200
Milano, Italy	Roche Diagnostics, Mannheim, Germany	Roche BRAHMS PCT	Electrochemiluminescence immunoassay (ECLIA)	30	0.02–100
Alessandria, Italy	Siemens Diagnostics, Marburg, Germany	Siemens BRAHMS PCT	One-step chemiluminescence immunoassay	100	0.02–75
Bassano del Grappa, Italy	Abbott Diagnostics Abbott Park, Illinois, U.S.A	Diazyme procalcitonin^a^	Latex-enhanced immunotubidimetric assay	20	0.16–56
Alessandria, Italy	Siemens Healthcare Diagnostics, Marburg, Germany	Diazyme procalcitonin^a^	Latex-enhanced immunotubidimetric assay	20	0.16–56
Milano, Italy	Roche Diagnostics, Mannheim, Germany	Diazyme procalcitonin^a^	Latex-enhanced immunotubidimetric assay	20	0.16–56
Parma, Italy	Beckman Coulter Inc., Brea, CA, USA	Diazyme procalcitonin^a^	Latex-enhanced immunotubidimetric assay	20	0.16–56

a Diazyme procalcitonin, Diazyme Laboratories, Poway, CA, USA.

Diazyme PCT (Diazyme Laboratories, Poway, CA, USA) is a latex-enhanced immunoturbidimetric assay, which has been developed for use on a wide array of laboratory analyzers capable of reading absorbance at 600 nm. According to the test procedure, the PCT present in the serum sample binds to specific anti-PCT antibodies coated on latex particles and trigger their agglutination. The turbidity is then measured at 600 nm and is directly proportional to the concentration of PCT in the sample. The final PCT value is calculated by interpolation of optical signal against a 6-point calibration curve. The amount of sample required for testing is 20 μL and the first test result is available in 10 min. In this study, Diazyme PCT was measured on four different clinical chemistry platforms, using the same assay protocol (Table 1). According to a previous analytical evaluation, the lowest measurable value and the total imprecision were found to be 0.16 ng/mL and 2.9–7.7%, respectively [13].

According to the manufacturers’ literature, the inter- and intra-assay imprecision (CV) of the other immunoassays used in this study is 2.0–3.8% and 3.2–9.5%, respectively for Liaison BRAHMS PCT; 1.9–4.6% and 3.6–7.0%, respectively for Vidas BRAHMS PCT; and 1.1–7.1% and 1.6–8.7%, respectively for Roche BRAHMS PCT. The manufacturer’s quoted total imprecision for Siemens BRAHMS PCT is <11%.

### 2.3 Statistical analysis

The results obtained using the different methods and analyzers were compared with those obtained with BRAHMS PCT Kryptor. Method comparison was based on Wilcoxon–Mann–Whitney paired rank sum test, Deming fit, Spearman’s correlation coefficient, Bland-Altman difference plots, receiver operating characteristics (ROC) curves, and agreement at three clinically relevant cut-offs for bacterial infections (i.e., 0.50 ng/mL, 2.0 ng/mL and 10 ng/mL) with kappa statistic. The statistical analysis was performed using Analyse-it (Analyse-it Software Ltd, Leeds, UK) and the level of statistical significance was set at *p*<0.05. The analytical evaluation was entirely based on pre-existing inpatient serum samples referred for routine PCT testing, so that informed patient consent was unnecessary. However, the study was carried out in accordance with the Declaration of Helsinki and under the terms of all relevant local legislation.

## 3. Results

**Fig. 2:**
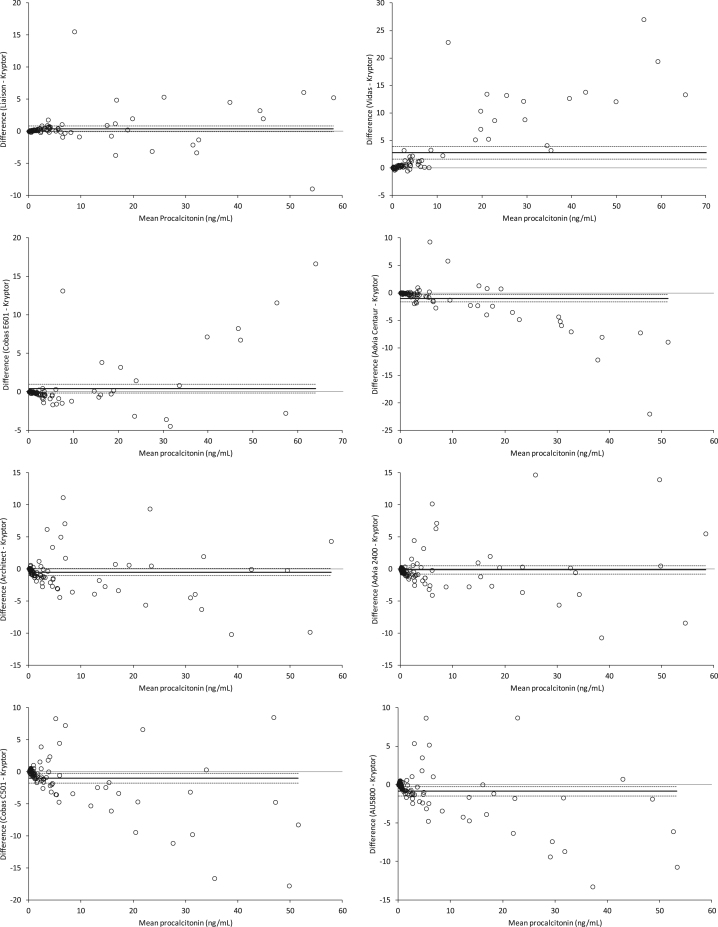
Bland and Altman plots of results obtained with different procalcitonin immunoassays (see Table 1 for details) compared to BRAHMS PCT Kryptor. The continuous line represents the mean bias, and the dotted lines are drawn at the 95% confidence interval (95% CI).

No significant decay of PCT immunoreactivity was observed after one week of storage at −70 °C, as attested by the negligible bias observed between fresh and frozen serum analyzed with BRAHMS PCT Kryptor (mean −0.05 ng/mL; 95% CI, −0.12 to 0.010 ng/mL; *p*=0.097). The distribution of PCT values obtained with different reagents and analyzers is shown in Fig. 1. Statistically significant differences compared to BRAHMS PCT Kryptor were observed for two BRAHMS-partnered PCT automated immunoassays (Vidas BRAHMS PCT and Advia Centaur BRAHMS PCT) and for three Diazyme PCT applications on Architect, Cobas C501 and AU5800 (Table 2). Nevertheless, the correlation between BRAHMS PCT Kryptor and the different methods and applications was always satisfactory, with correlation coefficients between 0.899 and 0.988 (all *p*<0.001). The mean bias was also limited and always lower than ±1.02 ng/mL, with the exception of Vidas BRAHMS PCT (2.70 ng/mL) (Table 2 and Fig. 2). Interestingly, this bias was mostly attributable to overestimation at PCT values higher than 10 ng/mL with Vidas BRAHMS PCT (mean bias 11.6 ng/mL, 95% CI 8.1–15.1 ng/mL for PCT values >10 ng/mL compared to mean bias of 0.6 ng/mL and 95% CI 0.04–1.19 ng/mL PCT for values <10 ng/mL) (Fig. 2). Other interesting information emerged from the comparison. Specifically, the bias between results obtained with BRAHMS PCT Kryptor and Advia Centaur BRAHMS PCT appeared to follow a specific linear pattern, characterized by overestimation of lower PCT values and underestimation of higher PCT concentration with the Advia immunoassay. Some outliers were also evident in almost every plot, but were more frequently observed for higher PCT values measured with Diazyme PCT applications (Fig. 2).

**Table 2:** Results obtained with procalcitonin immunoassays compared to BRAHMS PCT Kryptor.

	Values (ng/mL)^a^	*P* (Wilcoxon–Mann–Whitney)^b^	Spearman's correlation (*r*)^b^	Deming fit^b^	Mean bias and 95% CI (ng/mL)^b^
Kryptor	1.40 (0.35–5.37)	–	–	–	–
Liaison	1.63 (0.32–5.78)	0.052	0.986 (*p*<0.001)	*y*=1.02*x*+0.22	0.38 (−0.08 to 0.83)
Vidas	1.95 (0.33–6.40)	<0.001	0.979 (*p*<0.001)	*y*=1.40*x*−0.09	2.70 (1.55 to 3.86)
Cobas E601	1.30 (0.30–4.90)	0.085	0.988 (*p*<0.001)	*y*=1.12*x*−0.47	0.40 (−0.17 to 0.97)
Advia Centaur	1.35 (0.32–5.11)	0.001	0.984 (*p*<0.001)	*y*=0.80*x*+0.40	−1.02 (−1.67 to −0.36)
Diazyme (Architect)	1.27 (0.28–4.53)	0.038	0.899 (*p*<0.001)	*y*=0.95*x*−0.16	−0.51 (−1.07 to 0.06)
Diazyme (Advia 2400)	0.88 (1.15*–*4.46)	0.341	0.927 (*p*<0.001)	*y*=1.01*x*−0.23	−0.13 (−0.77 to 0.51)
Diazyme (Cobas C501)	0.90 (0.48*–*4.38)	0.004	0.899 (*p*<0.001)	*y*=0.85*x*+0.07	−1.01 (−1.76 to −0.26)
Diazyme (AU5800)	0.82 (0.42–4.82)	0.002	0.899 (*p*<0.001)	*y*=0.87*x*−0.04	−0.87 (−1.47 to −0.27)

a Median and interquartile range.

b Versus BRAHMS PCT Kryptor.

Overall, the agreement and the area under the curve (AUC) at the three clinically relevant cut-offs for bacterial infections were always satisfactory, with classification concordance between 83 and 98% at 0.50 ng/mL, between 90 and 97% at 2.0 ng/mL, and 98% at 10 ng/mL (Table 3). The direct comparison of data obtained with the Diazyme PCT assay on the four clinical chemistry analyzers yielded high correlation coefficients (between 0.952 and 0.976; all *p*<0.001), a mean bias always less than ±0.9 ng/mL, an acceptable classification agreement at the 0.5 ng/mL threshold (82–94%), and a high classification concordance at both the 2.0 ng/mL (97–99%) and 10 ng/mL (98–100%) cut-offs (Table 4).

**Table 3:** Agreement of results obtained with different procalcitonin immunoassays compared to BRAHMS PCT Kryptor at three diagnostic thresholds.

Procalcitonin cut-off (ng/mL)	Liaison	Vidas	Cobas E601	Advia Centaur	Diazyme (Architect)	Diazyme (Advia 2400)	Diazyme (Cobas C501)	Diazyme (AU5800)
0.5	97% (κ, 0.93) AUC, 0.96	97% (κ, 0.93) AUC, 0.98	98% (κ, 0.95) AUC, 0.99	95% (κ, 0.89) AUC, 0.95	84% (κ, 0.66) AUC, 0.86	86% (κ, 0.70) AUC, 0.89	86% (κ, 0.65) AUC; 0.81	83% (κ, 0.60) AUC, 0.80
2.0	96% (κ, 0.92) AUC, 0.96	94% (κ, 0.88) AUC, 0.95	97% (κ, 0.94) AUC, 0.97	96% (κ, 0.92) AUC, 0.96	90% (κ, 0.80) AUC, 0.89	91% (κ, 0.82) AUC, 0.91	92% (κ, 0.84) AUC, 0.92	91% (κ, 0.82) AUC, 0.90
10.0	98% (κ, 0.94) AUC, 0.97	98% (κ, 0.94) AUC, 0.99	98% (κ, 0.94) AUC, 0.97	98% (κ, 0.94) AUC, 0.96	98% (κ, 0.94) AUC, 0.96	98% (κ, 0.94) AUC, 0.96	98% (κ, 0.94) AUC, 0.95	98% (κ, 0.94) AUC, 0.98

AUC, area under the curve.

**Table 4:** Comparison of procalcitonin results obtained with Diazyme latex-enhanced immunotubidimetric assay on four different clinical chemistry platforms.

	Architect	Advia 2400	Cobas C501
Advia 2400	*r*=0.976 (*p*<0.001) Bias^a^, 0.37 ng/mL (0.05–0.70 ng/mL) Agreement^b^, 94%; 99%; 100%	–	–
Cobas C501	*r*=0.964 (*p*<0.001) Bias^a^, −0.50 ng/mL (−0.97 to –0.04 ng/mL) Agreement^b^, 84%; 98%; 98%	*r*=0.968 (*p*<0.001) Bias^a^, −0.88 ng/mL (−1.35 to −0.40 ng/mL) Agreement^b^, 82%; 99%; 98%	–
AU5800	*r*=0.958 (*p*<0.001) Bias^a^, −0.36 ng/mL (−0.65 to −0.07 ng/mL) Agreement^b^, 85%; 97%; 98%	*r*=0.962 (*p*<0.001) Bias^a^, −0.74 ng/mL (−1.18 to −0.29 ng/mL) Agreement^b^, 87%; 98%; 98%	*r*=0.952 (*p*<0.001) Bias^a^, 0.14 ng/mL (−0.22 to 0.50 ng/mL) Agreement^b^, 89%; 97%; 98%

a Mean bias and 95% Confidence Interval (95% CI).

b Classification agreement calculated at 0.5, 2.0 and 10 ng/mL cut-offs.

## 4. Discussion

The clinical value of measuring PCT for the diagnosis, follow-up and antibiotic guidance of severe bacterial infections has been confirmed in a large number of clinical trials [14], [15], [16], leading to the commercialization of many immunoassays to be used on conventional laboratory instrumentation. The current range of methods includes BRAHMS-partnered PCT automated immunoassays and a recently developed latex-enhanced immunoturbidimetric assay which can be applied to several analytical platforms. Although some studies have previously evaluated the individual performance of some of these techniques [8], [9], [10], [11], [12], [13], a comprehensive method comparison using identical serum samples has not been previously published to our knowledge. It is also noteworthy that no data are available on the direct comparison of Diazyme PCT results obtained on different clinical chemistry platforms.

The results of our multicenter evaluation suggest that the different reagents and techniques exhibit comparable performance at the three relevant diagnostic thresholds for bacterial infections. In particular, the agreement at the 2.0 and 10 ng/mL cut-offs, which reflect a moderate and high risk for progression to systemic inflammation, sever sepsis and septic shock, was excellent, displaying an overall classification concordance equal to or better than 90% with our reference method (BRAHMS PCT Kryptor) (Table 3). The agreement of data obtained using Diazyme PCT on four different clinical chemistry platforms was even better at these thresholds, ranging between 97% and 100% (Table 4). Regardless of the satisfactory correlation of values, a modest bias was still observed using different reagents and analyzers (Table 2), especially at PCT values higher than 10 ng/mL (Fig. 2). This was particularly evident when comparing BRAHMS PCT Kryptor with Vidas BRAHMS PCT and was mostly attributable to overestimation by the Vidas method at PCT serum concentration >10 ng/mL. Interestingly, a previous evaluation of Vidas BRAHMS PCT reported a lower bias against BRAHMS PCT Kryptor (i.e., 0.108 ng/mL), but an identical trend of overestimation in samples with PCT values >10 mg/L [10]. The overall agreement between BRAHMS PCT Kryptor and Vidas BRAHMS PCT at the 0.1, 0.25, 0.5 and 2 ng/mL thresholds in that study was however excellent (>96%), and comparable to that observed in our investigation (Table 3). The mean bias observed with the other reagents and instrumentation was modest (between −1.02 ng/ml and 0.40 ng/mL). This small difference does not substantially impact on the diagnosis of sepsis or classifying disease severity according to the current diagnostic cutoffs, but supports the hypothesis that the different methods cannot be used interchangeably for patient monitoring. Interestingly, the results obtained with the four Diazyme PCT applications were well correlated and substantially aligned with each other (Table 4), but they all exhibited a negative bias compared to BRAHMS PCT Kryptor, a fact that is probably attributable to the use of different calibrators. In this respect, the development and use of a common calibration material should be regarded as a viable proposition for improving agreement between methods.

## 5. Conclusions

The results of our investigation demonstrate that the methods and applications evaluated in this multicenter study are substantially aligned with BRAHMS PCT Kryptor, and can hence be used for predicting the risk of progression to systemic inflammation, severe sepsis and septic shock in patients with bacterial infections using the conventional PCT diagnostic thresholds. Nevertheless, the varying bias between by the different methods indicates that longitudinal patient monitoring should preferably always be carried out using the same immunoassay.

## Conflict of interest

None of the authors have any conflict of interest to report.
